# Treatment effectiveness of antibiotic therapy in Veterans with multidrug-resistant *Acinetobacter spp.* bacteremia

**DOI:** 10.1017/ash.2023.500

**Published:** 2023-12-12

**Authors:** Amanda Vivo, Margaret A. Fitzpatrick, Katie J. Suda, Geneva M. Wilson, Makoto M. Jones, Martin E. Evans, Charlesnika T. Evans

**Affiliations:** 1 Center of Innovation for Complex Chronic Healthcare (CINCCH), Edward Hines Jr. VA Medical Center, Hines, IL, USA; 2 Center of Innovation for Veteran Centered and Value Drive Care, Rocky Mountain Regional VA Medical Center, Aurora, CO, USA; 3 University of Colorado Anschutz Medical Campus, Aurora, CO, USA; 4 Center for Health Equity Research and Promotion, VA Pittsburgh Health Care System, Pittsburgh, PA, USA; 5 Department of Medicine, University of Pittsburgh School of Medicine, Pittsburgh, PA, USA; 6 Department of Preventive Medicine, Center for Health Services and Outcomes Research, Northwestern University Feinberg School of Medicine, Chicago, IL, USA; 7 Veterans Affairs Salt Lake City Health Care System, Salt Lake City, UT, USA; 8 Division of Epidemiology, Department of Internal Medicine, Spencer Fox Eccles School of Medicine, University of Utah, Salt Lake City, UT, USA; 9 VHA MRSA/MDRO Program Office, the National Infectious Diseases Service, Patient Care Services, VA Central Office and the Lexington VA Medical Center, Lexington, KY, USA; 10 Department of Internal Medicine, University of Kentucky School of Medicine, Lexington, KY, USA

## Abstract

**Objective:**

To describe antimicrobial therapy used for multidrug-resistant (MDR) *Acinetobacter spp.* bacteremia in Veterans and impacts on mortality.

**Methods:**

This was a retrospective cohort study of hospitalized Veterans Affairs patients from 2012 to 2018 with a positive MDR *Acinetobacter spp.* blood culture who received antimicrobial treatment 2 days prior to through 5 days after the culture date. Only the first culture per patient was used. The association between treatment and patient characteristics was assessed using bivariate analyses. Multivariable logistic regression models examined the relationship between antibiotic regimen and in-hospital, 30-day, and 1-year mortality. Generalized linear models were used to assess cost outcomes.

**Results:**

MDR *Acinetobacter spp.* was identified in 184 patients. Most cultures identified were *Acinetobacter baumannii (90%)*, 3% were *Acinetobacter lwoffii,* and 7% were other *Acinetobacter species.* Penicillins—β-lactamase inhibitor combinations (51.1%) and carbapenems (51.6%)—were the most prescribed antibiotics. In unadjusted analysis, extended spectrum cephalosporins and penicillins—β-lactamase inhibitor combinations—were associated with a decreased odds of 30-day mortality but were insignificant after adjustment (adjusted odds ratio (aOR) = 0.47, 95% CI, 0.21–1.05, aOR = 0.75, 95% CI, 0.37–1.53). There was no association between combination therapy vs monotherapy and 30-day mortality (aOR = 1.55, 95% CI, 0.72–3.32).

**Conclusion:**

In hospitalized Veterans with MDR *Acinetobacter spp*., none of the treatments were shown to be associated with in-hospital, 30-day, and 1-year mortality. Combination therapy was not associated with decreased mortality for MDR *Acinetobacter spp.* bacteremia.

## Introduction

*Acinetobacter* causes approximately 12,000 healthcare-associated infections annually in the United States and 63%–80% of these are multidrug-resistant (MDR).^1–3^ Carbapenem-resistant *Acinetobacter*, listed as a serious threat in the CDC’s 2019 Antibiotic Resistance Threat Report, are associated with approximately 8,500 cases and 700 deaths annually in the United States.^2^

MDR *Acinetobacter* infections are associated with increased mortality, reduced functional status at discharge, and need for mechanical ventilation, in comparison to those without MDR *Acinetobacter*.^4^ Timely and appropriate antimicrobial treatment is necessary to impede these adverse outcomes, but providers often need to initiate empiric therapy without final culture and susceptibility results.^5^ Treatment guides recommend monotherapy for mild illness; the Sanford Guide recommends cefepime, meropenem, or ampicillin-sulbactam as monotherapy for non-critical illness.^6^ The Infectious Disease Society of America (IDSA) guidelines recommend monotherapy with ampicillin-sulbactam, polymyxin B, or tetracyclines for mild carbapenem-resistant *Acinetobacter baumannii* (CRAB).^7^ Currently, ampicillin-sulbactam, carbapenems, tigecycline, and polymyxins are frequently used for MDR *Acinetobacter* infections, but increasing resistance limits available treatment options and illness severity may require a combination of antimicrobials.^8–10^ Additionally, a systematic review demonstrated superiority of combination treatment for severely ill patients with MDR *Acinetobacter* infection, as mortality was lowest among patients that received combination therapy compared to monotherapy.^11^ The Sanford Guide recommends a combination of polymyxin B, meropenem, and ampicillin-sulbactam for critically ill patients,^6^ while IDSA recommends combination therapy with high-dose ampicillin-sulbactam, minocycline, tigecycline, or polymyxin B for moderate to severe CRAB infections.^12^

Existing data are lacking to identify a preferred antibiotic treatment regimen for MDR *Acinetobacter* infections in current practice. Therefore, the objective of this study was to describe the treatments used for MDR *Acinetobacter spp.* bacteremia in a cohort of Veterans and assess the impact on in-hospital mortality, 30-day mortality, and 1-year mortality and costs.

## Materials and methods

### Study design, setting, and population

A retrospective cohort study was conducted using national VA medical encounter, pharmacy, and microbiology laboratory data from patients ≥18 years of age hospitalized at 134 VA facilities. The sample included patients from January 1, 2012 through December 31, 2018 with a blood culture positive for MDR *Acinetobacter spp.* who received treatment within the time frame of 2 days prior through 5 days after the culture date.

### Data sources and definitions

Data were extracted from the VA Corporate Data Warehouse (CDW), which is a national repository that includes clinical and administrative data from the Veterans Health Administration. These data are updated on a continual basis and were used to obtain patient demographics, clinical setting at the time of culture, diagnoses and procedures, microbiology data, comorbidities, previous antibiotic exposure, medications, and facility characteristics. Microbiology data were used to identify *Acinetobacter spp.* from blood cultures. MDR *Acinetobacter spp.* was defined as any isolate that was resistant or intermediate in susceptibility to three or more of the following antimicrobial groups: aminoglycosides, carbapenems, fluoroquinolones, extended-spectrum cephalosporins, folate pathway inhibitors, penicillins—β-lactamase inhibitor combinations, polymyxins, and tetracyclines.^13^ Antibiotic susceptibility testing was performed by each VA laboratory according to their local protocol.

Pharmacy data were used to identify antibiotics prescribed during hospitalization. Only antibiotics with likely spectrum of activity against *Acinetobacter* (Supplemental Table 1) and prescribed within the −2 through +5 days window from the culture date were included. Monotherapy was defined as receipt of one antibiotic and combination therapy was defined as receipt of more than one antibiotic for any duration, within the time fame. Intracellular plus extracellular combination therapy was defined as treatment that included one antimicrobial agent that targets the components within the cell plus an agent that targets the cell wall. Aminoglycosides, rifampin, fluoroquinolones, glycylcyclines, sulfonamides, and tetracyclines were defined as intracellular agents. Penicillins—β-lactamase inhibitor combinations, carbapenems, extended spectrum cephalosporins, and polymyxins—were defined as extracellular agents. Fully adequate treatment was defined as receipt of only antibiotics to which the isolate was non-resistant (susceptible or intermediate). Partial adequate treatment was defined as receipt of at least one antibiotic to which the isolate was non-resistant. Inadequate treatment was defined as receipt of no antibiotics to which the isolate was non-resistant. Cultures within 30 days of each other were removed if organism and treatment were identical and only the first culture per patient was included.

Patient characteristics of gender, age, race, ethnicity, comorbidities, and previous healthcare exposure (previous ICU admission, past immunocompromising conditions, mechanical ventilation, previous antibiotic exposure, and previous admission) were also collected. Previous healthcare exposures were defined as occurring in the previous 90 days before the positive culture. Comorbidities were identified by ICD-10 codes in the last 365 days prior to an MDR *Acinetobacter spp.* culture and used to calculate the Charlson comorbidity index.^14^ Previous antibiotic exposures were defined as antibiotics prescribed in the past 90 days before the positive culture. Additionally, length of stay (LOS) post-culture was collected.

Outcome variables collected included in-hospital mortality, 30-day mortality, and 1-year mortality. In-hospital mortality was defined as death that occurred while in the hospital during that admission, while 30-day and 1-year mortality were defined as deaths that occurred within 30 days and 365 days after the culture date. Secondary outcomes included infectious disease as cause of death attributable to infectious disease, which was obtained from the VA and Department of Defense Suicide Data Repository and healthcare costs, which were obtained from the National Data Extracts Managerial Cost Accounting Office and included pharmacy, inpatient, outpatient, total fee, and total costs at 30 days and 1 year after culture.^15,16^ Total costs were the sum of inpatient, outpatient, and pharmacy costs.

To validate the MDR definition and the ICD-10 codes for infections, medical records were reviewed for 61 blood cultures. Agreement between MDR definition for these cultures using CDW data and medical record review was 100%, while agreement for true *Acinetobacter spp.* infection was 95%. Reasons for non-agreement were infectious disease notes from medical record indicating the Acinetobacter was a contaminant and not a true blood infection.

### Statistical analyses

Descriptive and bivariate statistics were used to summarize patient demographics, medical characteristics, previous healthcare exposure, microbiology data, facility characteristics, treatments received, and outcomes. Chi-square and Fisher’s exact tests were used to assess the relationship between each treatment type and medical, demographic, and facility-level variables. Unadjusted and adjusted logistic regression modeling was used to assess antibiotic regimen and in-hospital, 30-day, and 1-year mortality. Generalized linear models, unadjusted and adjusted, were also fit for the cost analyses. Variables were included in the adjusted models if they were significant in the unadjusted analyses or if they were previously cited risk factors, with the most parsimonious models selected. A *p*-value of ≤0.05 was considered statistically significant for all analyses. Sensitivity analyses for costs were also conducted, excluding Veterans who died at 30 days and 1 year. Statistical analyses were conducted using SAS, version 9.4 (SAS Institute) and STATA/MP version 14.2 (StataCorp LLC).

## Results

One-hundred and eighty-four patients were identified with blood cultures during hospitalization that grew MDR *Acinetobacter spp.* and received antimicrobial treatment based on study definition. Ninety percent of cultures identified were *Acinetobacter baumannii*, 3% were *Acinetobacter lwoffii,* and 7% were other *Acinetobacter species.* Most patients were older (mean age = 67, standard deviation = 12.7), white, and non-Hispanic males. Prior hospital admission (77.2%) and antibiotic exposure (90.8%) in the previous 90 days were common. From 2012 to 2018, these treatments were commonly used in accordance with recommendations from the Sanford Guide: carbapenems (51.6%), aminoglycosides (29.9%), and extended spectrum cephalosporins (31.5%) (Table 1). Supplemental Table 1 shows specific antibiotics included in these classes. Penicillins—β-lactamase inhibitor combinations—were also frequently prescribed (51.1%). Half of cultures received combination anti-*Acinetobacter* therapy (50.0%), and 50.0% received monotherapy (Table 1). Extended spectrum cephalosporins were most commonly used as monotherapy (46.6%), followed by carbapenems (40.0%) and then penicillins—β-lactamase inhibitor combinations (39.4%). Most combination therapies included an aminoglycoside (78.2%), a polymyxin (67.7%), or a penicillin + β-lactamase inhibitor combination (60.6%). Thirty-seven percent of patients received fully adequate treatment, 50% partially adequate, and 13% inadequate treatment. Patients who received aminoglycosides and extended spectrum cephalosporins were more likely to have partial and fully adequate treatment (Table 1).

**Table 1.** Demographics and characteristics by select treatments for bloodstream infection with MDR *Acinetobacter spp.*

	Total	Carbapenems	Aminoglycosides	Polymyxins	Extended spectrum cephalosporins	Penicillins—β-lactamase inhibitor combinations	Combination therapy
	*N* (%) = 184	*N* (%) = 95 (51.6)	*N* (%) = 55 (29.9)	*N* (%) = 31 (16.9)	*N* (%) = 58 (31.5)	*N* (%) = 94 (51.1)	*N* (%) = 92 (50.0)
**Age**							
18–49	9 (4.9)	3 (3.2)	0 (0)	2 (6.5)	1 (1.7)	6 (6.4)	5 (5.4)
50–64	65 (35.3)	35 (36.8)	22 (40.0)	8 (25.8)	25 (43.1)	33 (25.1)	35 (38.0)
65+	110 (59.8)	57 (60.0)	33 (60.0)	21 (67.7)	32 (55.2)	55 (58.5)	52 (56.5)
**Race**							
White	101 (54.9)	59 (62.1)	26 (47.3)	16 (51.6)	32 (55.2)	50 (53.2)	51 (55.4)
Black	66 (35.9)	28 (29.5)	23 (41.8)	12 (38.7)	22 (37.9)	35 (37.2)	35 (38.0)
Other/missing	17 (9.2)	8 (8.4)	6 (10.9)	3 (9.7)	4 (6.9)	9 (9.6)	6 (6.5)
**Ethnicity**		*		***		**	
Non-Hispanic	151 (82.1)	72 (75.8)	45 (81.8)	18 (58.1)	46 (79.3)	85 (90.4)	74 (80.4)
Hispanic	33 (17.9)	23 (24.2)	10 (18.2)	13 (41.9)	12 (20.7)	9 (9.6)	18 (19.6)
**Charlson mean (SD)**	4.2 (3.2)	4.28 (3.51)	4.38 (3.25)	3.71 (4.11)	4.21 (2.98)	4.33 (2.95)	4.48 (3.36)
**Gagne score mean (SD)**	15.0 (23.6)	13.99 (25.19)	27.96 (28.33)	5.87 (10.21)***	11.76 (16.79)	16.31 (21.20)	17.13 (27.19)
**Immunocompromised in <90 days**	0 (0)	0 (0)	0 (0)	0 (0)	0 (0)	0 (0)	0 (0)
**Admission in <90 days**	142 (77.2)	78 (82.1)	40 (72.7)	25 (80.7)	43 (74.1)	69 (73.4)	71 (77.2)
**ICU in <90 days**	23 (12.5)	13 (13.7)	7 (12.7)	1 (3.2)	9 (15.5)	12 (12.8)	11 (12.0)
**Antibiotic use <90 days**	167 (90.8)	89 (93.7)	50 (90.9)	30 (96.8)	49 (84.5)	83 (88.3)	83 (90.2)
**Mech. vent. <90 days**	57 (31.0)	32 (33.7)	18 (32.7)	13 (41.9)	16 (27.6)	22 (23.4) *	23 (25.0)
**LOS median, mean (SD)**	18.0, 49.0 (74.9)	18.0, 58.6 (91.3)	18.0, 50.5 (72.1)	20.0, 31.4 (28.5)	25.5, 61.2 (88.9)	24.0, 55.6 (83.4)	24.0, 62.8 (83.6)
**Treatment adequacy**			**		**		
Total adequacy	69 (37.5)	27 (28.4)	16 (29.1)	11 (35.5)	10 (17.2)	34 (36.2)	22 (23.9)
Partial adequacy	91 (49.5)	57 (60.0)	37 (67.3)	20 (64.5)	39 (67.2)	52 (55.3)	64 (69.6)
Inadequate	24 (13.0)	11 (11.6)	2 (3.6)	0 (0)	9 (15.5)	8 (8.5)	6 (6.5)
**Mono/combo therapy**		**	***	*		**	
Monotherapy	92 (50.0)	38 (40.0)	12 (21.8)	10 (32.3)	27 (46.6)	37 (39.4)	\\\
Combination therapy	92 (50.0)	57 (60.0)	43 (78.2)	21 (67.7)	31 (52.4)	57 (60.6)	\\\

Those who received treatments are compared to those who did not receive the specified treatment; *= significant at 0.05; **= significant at 0.01; ***= significant at 0.0001.

The median LOS for these patients was 18 days. Half (50.5%) of patients died in hospital, with 16.5% of these attributable to infectious disease. Forty-four percent of patients died within 30 days, with 20.0% of these deaths attributable to infectious disease. Two-thirds (67.9%) of the population died within 1 year where 15.3% of these deaths were attributable to infectious disease.

In unadjusted analysis, extended spectrum cephalosporins and penicillins—β-lactamase inhibitor combinations—were associated with a decreased odds of 30-day mortality but were insignificant after adjustment (adjusted odds ratio (aOR) = 0.47, 95% CI, 0.21–1.05, aOR = 0.75, 95%CI, 0.37–1.53). After adjustment, no antibiotic therapy was found to be significantly associated with an increase or decrease in 30-day mortality (Table 2 and Supplemental Table 2), in-hospital mortality (Table 3 and Supplemental Table 3), and 1-year mortality. Combination therapy was also not associated with in-hospital, 30-day, and 1-year mortality in adjusted analyses.

**Table 2.** Unadjusted and adjusted logistic regression models assessing the association between antibiotic therapy and 30-day mortality for bloodstream infection with MDR *Acinetobacter spp.*

Antibiotic therapy	Unadjusted		Adjusted	
	OR (95%CI)	*p*-value	OR (95%CI)	*p*-value
Carbapenems	1.58 (0.88–2.85)	0.1246	1.96 (0.96–4.02)	0.0649
Aminoglycosides	1.34 (0.71–2.52)	0.3664	1.69 (0.76–3.72)	0.1949
Polymyxins	1.98 (0.9–4.33)	0.0800	2.46 (0.94–6.44)	0.0669
Fluoroquinolones	0.91 (0.44-1.87)	0.7894	1.16 (0.47-2.84)	0.7479
Ex. spec. cephalosporins	**0.5 (0.26**–**0.96)**	**0.0382**	0.47 (0.21–1.05)	0.0643
Penicillins—β-lactamase inhibitor combinations	**0.52 (0.29**–**0.94)**	**0.0291**	0.75 (0.37–1.53)	0.4296
Tetracyclines	0.62 (0.15–2.57)	0.5112	0.96 (0.16–5.63)	0.9613
Sulfonamides	0.54 (0.16–1.83)	0.3242	0.37 (0.08–1.8)	0.2185
Glycylcyclines	1.29 (0.4–4.17)	0.6668	2.25 (0.55–9.17)	0.2593
Combination therapy	0.73 (0.41–1.32)	0.2991	1.55 (0.72–3.32)	0.2591
Intra+extra therapy	1.19 (0.66–2.13)	0.5645	1.65 (0.76–3.6)	0.2067

Significant associations are shown in bold.

**Table 3.** Unadjusted and adjusted logistic regression models assessing the association between antibiotic therapy and in-hospital mortality for bloodstream infection with MDR *Acinetobacter spp.*

Antibiotic therapy	Unadjusted		Adjusted	
	OR (95%CI)	*p*-value	OR (95%CI)	*p*-value
Carbapenems	1.69 (0.94–3.03)	0.0783	1.65 (0.85–3.18)	0.1367
Aminoglycosides	1.26 (0.67–2.37)	0.4787	1.24 (0.6–2.55)	0.5587
Polymyxins	1.99 (0.89–4.44)	0.0917	1.96 (0.81–4.73)	0.1354
Fluoroquinolones	0.97 (0.48–1.99)	0.9400	1.15 (0.5–2.62)	0.7389
Ex. spec. cephalosporins	0.65 (0.35–1.21)	0.1720	0.59 (0.3–1.16)	0.1268
Penicillins—β-lactamase inhibitor combinations	0.62 (0.34–1.11)	0.1049	0.74 (0.39–1.4)	0.3531
Tetracyclines	0.77 (0.2–2.98)	0.7081	0.89 (0.21–3.7)	0.8713
Sulfonamides	1.62 (0.51–5.15)	0.4144	1.58 (0.44–5.71)	0.4822
Glycylcyclines	0.68 (0.21–2.23)	0.5268	0.58 (0.16–2.04)	0.3925
Combination therapy	1.14 (0.64–2.03)	0.6583	1.44 (0.71–2.95)	0.3158
Intra+extra therapy	1.30 (0.73–2.32)	0.3758	1.35 (0.66–2.76)	0.4114

**Figure 1.**
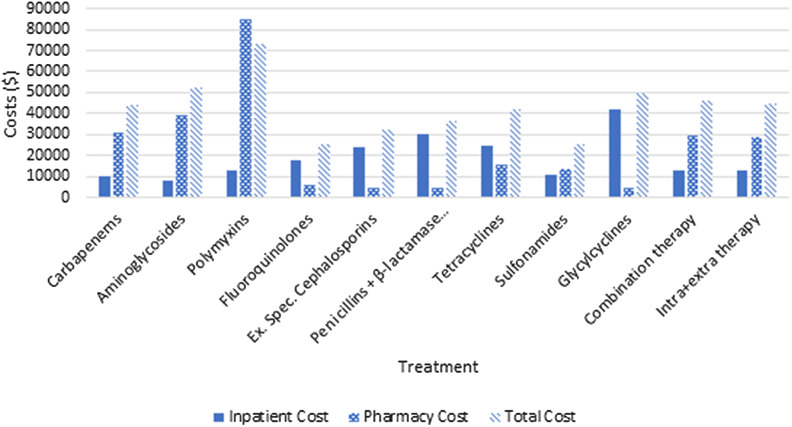
Mean inpatient, pharmacy, and total costs at 30 days for treatment of bloodstream infection with MDR *Acinetobacter spp.* stratified by antibiotic class.

Overall, the average total cost at 30 days for Veterans with MDR *Acinetobacter spp.* bacteremia was $39,712 per patient (standard deviation: $55,781). Average total costs at 1 year were highest in those treated with tetracyclines ($341,486) and lowest in those receiving fluoroquinolones ($162,588). Those treated with polymyxins had the highest average total cost ($73,116) and fluoroquinolones had the lowest ($25,135) (Figure 1). Adjusted generalized linear models for cost found an association between aminoglycoside and polymyxin use and increased total cost at 30 days compared to no usage of aminoglycosides or polymyxins (Table 4). Carbapenem and polymyxin use were associated with higher total pharmacy cost, while extended spectrum cephalosporin and penicillins—β-lactamase inhibitor combinations use—were associated with lower pharmacy costs at 30 days compared to no usage of these antibiotics. Penicillins—β-lactamase inhibitor combinations and glycylcyclines use—were associated with higher inpatient costs at 30 days compared to no usage of these antibiotics. However, in the sensitivity analysis excluding patients that died within 30 days, there were no antibiotic therapies significantly associated with inpatient costs. Extended spectrum cephalosporin use was no longer associated with pharmacy costs, while combination therapy was associated with higher pharmacy cost. In the sensitivity analysis, aminoglycoside and polymyxin use were no longer associated with total cost at 30 days, but penicillins—β-lactamase inhibitor combinations—were associated with higher total cost in 30 days and sulfonamides were associated with lower total cost in 30 days (Supplemental Table 4).

**Table 4.** Adjusted generalized linear models for cost at 30 days for bloodstream infection with MDR *Acinetobacter spp*. stratified by antibiotic regimen

Antibiotic therapy	Adjusted					
	Inpatient cost		Pharmacy cost		Total cost	
	Model OR (95%CI)	*p*-value	Model OR (95%CI)	*p*-value	Model OR (95%CI)	*p*-value
Carbapenems	Model not significant		**0.68 (0.10**–**1.26)**	**0.022**	0.11 (−0.28 to 0.51)	
Aminoglycosides	Model not significant		0.41 (−0.18 to 1.00)		**0.47 (0.04**–**0.90)**	**0.032**
Polymyxins	Model not significant		**2.63 (1.58**–**3.69)**	**0.000**	**0.55 (0.06**–**1.05)**	**0.028**
Fluoroquinolones	Model not significant		Model not significant		Model not significant	
Ex. spec. cephalosporins	Model not significant		−**0.91 (**−**1.50 to** −**0.40)**	**0.001**	Model not significant	
Penicillins—β-lactamase inhibitor combinations	**16.70 (14.66**–**18.74)**	**0.000**	−**1.02 (**−**1.68 to** −**0.37)**	**0.002**	Model not significant	
Tetracyclines	Model not significant		Model not significant		Model not significant	
Sulfonamides	Model not significant		Model not significant		Model not significant	
Glycylcyclines	**7.57 (0.90**–**14.26)**	**0.026**	Model not significant		Model not significant	
Combination therapy	Model not significant		Model not significant		Model not significant	
Intra+extra therapy	Model not significant		Model not significant		Model not significant	

Significant associations are shown in bold.

## Discussion

In this national analysis of Veterans with positive MDR *Acinetobacter spp.* blood cultures during hospitalization, carbapenems and penicillins—β-lactamase inhibitor combinations—were the most frequently used antibiotics. This is consistent with current literature, as they are the agents of choice for *Acinetobacter* infections.^8,17^ However, the benefit of combination therapy for MDR *Acinetobacter* infections was not supported in this study. Given limited clinical data supporting the effectiveness of any single antibiotic agent, both the Sanford Guide and the IDSA guidelines recommend combination therapy including high-dose ampicillin-sulbactam for severe MDR *Acinetobacter spp.* infection.^6,12^ However, prior studies have found limited improved clinical outcomes with combination therapy.^18–21^ Our results provide additional comparative effectiveness demonstrating a lack of benefit to combination therapy given within −2 through +5 days from the culture date.

Use of penicillins—β-lactamase inhibitor combinations—was associated with decreased odds of 30-day mortality; however, associations were not significant after adjustment. This may have been due to the lack of granularity in the antibiotic classes. Ampicillin-sulbactam is a recommended treatment in IDSA and Sanford guidelines, but this was not separated from the penicillins—β-lactamase inhibitor combinations class. However, penicillins—β-lactamase inhibitor combination use—were associated with lower pharmacy costs at 30 days in both cost analyses suggesting that mortality did not lead to lower costs. Carbapenems were associated with increased pharmacy costs at 30 days in the cost analyses but were not significantly associated with survival, despite meropenem being recommended treatment. This again may have been due to meropenem not being separated out of the carbapenem class. Interestingly, polymyxins were not commonly prescribed, nor were they associated with a reduction in mortality in this study, despite literature illustrating their successful use against other drug-resistant gram-negative bacteria.^8,17,22^ This may be a result of clinicians erring on the side of caution, as the study population was older and had comorbidities involving renal impairment, and nephrotoxicity and neurotoxicity are concerns when using polymyxins.^17^ As literature has found polymyxins to be successful against drug-resistant gram-negative bacteria and *Acinetobacter*, it is necessary to further explore this relationship.^8,17,22,23^

In-hospital mortality was higher than 30-day mortality but deaths that occurred within 30 days had a greater percentage of mortality attributable to infectious disease. Additionally, patients who died during hospitalization also had longer LOS, which may be a result of other comorbidities contributing to longer LOS.^24,25^ Patients who died within 30 days had shorter LOS and higher mortality attributable to infectious disease suggesting that death from infectious disease may have contributed to the shorter LOS.

This study had several limitations. First, this analysis only included VA data and may not be representative of MDR *Acinetobacter baumannii.* in non-VA populations. Second, this analysis only included patients who received treatment within −2 through +5 days of culture date, which may have resulted in missing patients who may have died before treatment, had delayed treatment, or received treatment longer than 5 days. Third, treatment received more than 5 days post-culture was not captured; thus, full duration of treatment was not a factor in the outcome or cost analysis, which may have resulted in underestimated costs in severely ill patients. Additionally, the identification and reporting of bacterial susceptibilities could only be determined based on antibiotics that were tested in the microbiology panels. This may have led to an overestimated adequacy of treatment. Additionally, only 33% of the cohort was chart reviewed, which may not have sufficiently validated MDR and ICD-10 definitions for the cohort. Another limitation is the data time frame was prior to FDA approval of some of the newer medications that would be used to treat MDR *Acinetobacter spp.* bacteremia, such as cefiderocol. Finally, patients may have several antibiotic changes during the treatment window and antibiotics were identified in their antibiotic classes and were not individually identified. Thus, differentiating the impact of a single antibiotic treatment was not possible.

Based on our study results, penicillins—β-lactamase inhibitor combinations—were frequently prescribed and were associated with higher mortality in the unadjusted analysis. However, after adjustment there were no associations between treatment and mortality. In addition, these results support the lack of benefit to combination therapy for treatment of MDR *Acinetobacter spp.* bacteremia, and as there was no treatment associated with decreased mortality and lower total costs, treatment regimens can be chosen based on clinical success rather than costs.

## Supporting information

Vivo et al. supplementary material Vivo et al. supplementary material

## Supplementary material

For supplementary material accompanying this paper visit http://doi.org/10.1017/ash.2023.500. click here to view supplementary material

## Research transparency and reproducibility

We are committed to collaborating and sharing these data to maximize their value to improve Veterans and others’ health and health care, to the greatest degree consistent with current Veterans Administration regulations and policy. We can provide access to the programming code used to identify the sample and conduct analyses. We cannot provide a link to the database directly as it would compromise patients’ anonymity, and permissions from VA are needed to obtain the data.
