# Supplementary material for: Treatment effectiveness of antibiotic therapy in Veterans with multidrug-resistant Acinetobacter spp. bacteremia
Source: Antimicrob Steward Healthc Epidemiol. 2023 Dec 12;3(1):e230. doi: 10.1017/ash.2023.500 (PMC10753468; doi:10.1017/ash.2023.500)
Supplement: Vivo et al. supplementary material [file S2732494X23005004sup001.docx]

Supplemental Table 1. Antibiotic treatments evaluated in this study with spectrum of activity against MDR *Acinetobacter spp.*

| Antimicrobial Class | Agents^a^ |
| --- | --- |
| Polymyxins | Polymyxin B  Colistin |
| Penicillins + B-lactamase inhibitors | Piperacillin-tazobactam  Ampicillin-sulbactam |
| Extended spectrum cephalosporins | Cefepime  Ceftazidime |
| Carbapenems | Imipenem  Meropenem  Doripenem |
| Glycylcyclines | Tigecycline |
| Aminoglycosides | Amikacin  Tobramycin  Gentamicin |
| Tetracyclines | Doxycycline  Minocycline |
| Fluoroquinolones | Levofloxacin  Ciprofloxacin |
| Sulfonamides | Trimethoprim/sulfamethoxazole |
| Antimycobacterials\Rifamycins | Rifampin^b^ |

^a^List derived from international consensus definition for MDR *Acinetobacter* and antibiotic susceptibility results for this cohort of cultures^14^

^b^Only evaluated as part of combination therapy

Supplemental Table 2. Adjusted logistic regression models assessing the association between antibiotic therapy and 30-day mortality for Bloodstream Infection with MDR *Acinetobacter sp*.

|  | Total  N (%)=184 | Carbapenems  N (%) = 95 (51.6) | Aminoglycosides  N (%) = 55 (29.9) | Polymyxins  N (%) = 31 (16.9) | Extended Spectrum Cephalosporins  N (%) = 58 (31.5) | Penicillins - β-lactamase inhibitor combinations  N (%) = 94 (51.1) | Combination Therapy  N (%) = 92 (50.0) |
| --- | --- | --- | --- | --- | --- | --- | --- |
| **Not receiving specific antibiotic** |  | Reference | Reference | Reference | Reference | Reference | Reference |
| **Specific antibiotic** |  | 1.96 (0.96-4.02) | 1.69 (0.76-3.72) | 2.46 (0.94-6.44) | 0.47 (0.21-1.05) | 0.75 (0.37-1.53) | 1.55 (0.72-3.32) |
| **Age** |  |  |  |  |  |  |  |
| 18-49 | Reference | Reference | Reference | Reference | Reference | Reference | Reference |
| 50-64 | 5 (0.59-42.42) | 7.69 (0.84-69.91) | 7.93 (0.84-74.59) | 9.69 (1.05-89.62)* | 11.7 (1.22-112.12)* | 8.76 (0.94-81.55) | 9.52 (1.03-88.29)* |
| 65+ | 8 (0.97-66.13) | 8.70 (1-75.36)* | 9.31 (1.05-82.67)* | 10.47 (1.2-91.42)* | 12.23 (1.36-110.23)* | 10.27 (1.17-90.43)* | 10.94 (1.24-96.39)* |
| **Race** |  |  |  |  |  |  |  |
| White | Reference | Reference | Reference | Reference | Reference | Reference | Reference |
| Black | 0.67 (0.36-1.27) | \ | \ | \ | \ | \ | \ |
| Other/Missing | 0.98 (0.35-2.75) | \ | \ | \ | \ | \ | \ |
| **Ethnicity** |  |  |  |  |  |  |  |
| Non-Hispanic | Reference | \ | \ | \ | \ | \ | \ |
| Hispanic | 2.27 (1.05-4.9)* | \ | \ | \ | \ | \ | \ |
| **Charlson** | 1.09 (0.99-1.19) | 1.11 (0.99-1.24) | 1.1 (0.99-1.23) | 1.12 (1-1.25) | 1.1 (0.98-1.23) | 1.11 (0.99-1.24) | 1.1 (0.98-1.23) |
| **Gagne Score** | 1.01 (0.99-1.02) | \ | \ | \ | \ | \ | \ |
| **Admission in <90 days** | 1.56 (0.77-3.19) | \ | \ | \ | \ | \ | \ |
| **ICU in <90 days** | 0.51 (0.2-1.32) | \ | \ | \ | \ | \ | \ |
| **Antibiotic Use <90days** | 2 (0.68-5.94) | \ | \ | \ | \ | \ | \ |
| **Mech. Vent. <90 days** | 2.8 (1.47-5.34)** | 2.88 (1.33-6.23)** | 2.89 (1.35-6.19)** | 2.78 (1.29-6)** | 2.94 (1.36-6.35)** | 2.74 (1.27-5.9)* | 3.12 (1.44-6.77)** |
| **LOS** | 0.98 (0.96-0.99)*** | 0.97 (0.96-0.99)*** | 0.97 (0.96-0.98)*** | 0.97 (0.96-0.98)*** | 0.97 (0.96-0.99)*** | 0.97 (0.96-0.99)*** | 0.97 (0.96-0.98)*** |
| **Treatment Adequacy** |  |  |  |  |  |  |  |
| Total Adequacy | 0.55 (0.21-1.41) | 0.66 (0.21-2.1) | 0.59 (0.19-1.85) | 0.55 (0.17-1.75) | 0.5 (0.15-1.64) | 0.66 (0.21-2.07) | 0.61 (0.2-1.92) |
| Partial Adequacy | 0.49 (0.2-1.22) | 0.39 (0.13-1.23) | 0.37 (0.12-1.15) | 0.35 (0.11-1.11) | 0.43 (0.14-1.34) | 0.47 (0.15-1.45) | 0.36 (0.11-1.16) |
| Inadequate | 24 (13.0) | Reference | Reference | Reference | Reference | Reference | Reference |
| **Mono / Combo Therapy** |  |  |  |  |  |  |  |
| Monotherapy | Reference | Reference | Reference | Reference | Reference | Reference | \\\ |
| Combination therapy | 0.73 (0.41-1.32) | \ | \ | \ | \ | \ | \\\ |

Those who received treatments are compared to those who did not receive the specified treatment; *= significant at 0.05; **= significant at 0.01 ***= significant at 0.0001

Supplemental Table 3. Adjusted logistic regression models assessing the association between antibiotic therapy and in-hospital mortality for Bloodstream Infection with MDR *Acinetobacter sp*.

|  | Total  N (%)=184 | Carbapenems  N (%) = 95 (51.6) | Aminoglycosides  N (%) = 55 (29.9) | Polymyxins  N (%) = 31 (16.9) | Extended Spectrum Cephalosporins  N (%) = 58 (31.5) | Penicillins - β-lactamase inhibitor combinations  N (%) = 94 (51.1) | Combination Therapy  N (%) = 92 (50.0) |
| --- | --- | --- | --- | --- | --- | --- | --- |
| **Not receiving specific antibiotic** |  | Reference | Reference | Reference | Reference | Reference | Reference |
| **Specific antibiotic** |  | 1.65 (0.85-3.18) | 1.24 (0.6-2.55) | 1.96 (0.81-4.73) | 0.59 (0.3-1.16) | 0.74 (0.39-1.4) | 1.44 (0.71-2.95) |
| **Age** |  |  |  |  |  |  |  |
| 18-49 | Reference | Reference | Reference | Reference | Reference | Reference | Reference |
| 50-64 | 3.19 (0.62-16.54) | 4.43 (0.78-25.09) | 4.41 (0.77-25.16) | 4.23 (0.74-24.22) | 4.63 (0.82-26.05) | 3.66 (0.66-20.43) | 5.07 (0.89-29.08) |
| 65+ | 4.2 (0.83-21.13) | 5.73 (1.04-31.56)* | 5.81 (1.05-32)* | 5.91 (1.06-33.06)* | 6.32 (1.16-34.53)* | 5.41 (0.99-29.52) | 6.66 (1.19-37.14)* |
| **Race** |  |  |  |  |  |  |  |
| White | Reference | Reference | Reference | Reference | Reference | Reference | Reference |
| Black | 0.89 (0.48-1.65) | 0.96 (0.49-1.89) | 0.87 (0.44-1.72) | 0.87 (0.45-1.69) | 0.88 (0.45-1.7) | 0.89 (0.46-1.72) | 0.88 (0.45-1.74) |
| Other/Missing | 0.26 (0.08-0.84)* | 0.18 (0.05-0.64)** | 0.16 (0.04-0.57)** | 0.19 (0.05-0.68)* | 0.19 (0.05-0.65)** | 0.2 (0.06-0.7)* | 0.17 (0.05-0.61)** |
| **Ethnicity** |  |  |  |  |  |  |  |
| Non-Hispanic | Reference | \ | \ | \ | \ | \ | \ |
| Hispanic | 1.64 (0.76-3.54) | \ | \ | \ | \ | \ | \ |
| **Charlson** | 1.01 (0.92-1.1) | 0.99 (0.89-1.09) | 0.97 (0.87-1.07) | 0.99 (0.9-1.1) | 1 (0.9-1.1) | 0.99 (0.9-1.09) | 0.96 (0.87-1.07) |
| **Gagne Score** | 1 (0.99-1.02) | \ | \ | \ | \ | \ | \ |
| **Admission in <90 days** | 1.16 (0.58-2.32) | \ | \ | \ | \ | \ | \ |
| **ICU in <90 days** | 0.48 (0.19-1.19) | \ | \ | \ | \ | \ | \ |
| **Antibiotic Use <90days** | 3.71 (1.16-11.84)* | \ | 3.65 (1.06-12.59)* | \ | \ | \ | 3.73 (1.07-12.99)* |
| **Mech. Vent. <90 days** | 2.93 (1.51-5.67)** | 3 (1.47-6.14)** | 2.61 (1.26-5.41)** | 3.19 (1.58-6.47)** | 3.36 (1.66-6.79)** | 3.17 (1.56-6.47)** | 2.74 (1.31-5.72)** |
| **LOS** | 1 (0.99-1) | 1 (0.99-1)* | 1 (0.99-1)* | \ | \ | \ | 0.99 (0.99-1)* |
| **Treatment Adequacy** |  |  |  |  |  |  |  |
| Total Adequacy | 0.55 (0.21-1.41) | 0.57 (0.2-1.6) | 0.53 (0.19-1.49) | 0.51 (0.19-1.41) | 0.47 (0.17-1.32) | 0.58 (0.21-1.6) | 0.53 (0.19-1.49) |
| Partial Adequacy | 0.83 (0.34-2.07) | 0.79 (0.29-2.17) | 0.8 (0.29-2.24) | 0.74 (0.28-1.97) | 0.88 (0.33-2.35) | 0.91 (0.34-2.41) | 0.74 (0.26-2.09) |
| Inadequate | Reference | Reference | Reference | Reference | Reference | Reference | Reference |
| **Mono / Combo Therapy** |  |  |  |  |  |  |  |
| Monotherapy | Reference | Reference | Reference | Reference | Reference | Reference | \\\ |
| Combination therapy | 1.14 (0.64-2.03) | \ | \ | \ | \ | \ | \\\ |

Significant associations are shown in bold

Supplemental Table 4: Sensitivity analysis of adjusted Generalized Linear Models for Cost at 30-days for Bloodstream Infection with MDR *Acinetobacter spp.* stratified by antibiotic regimen

|  | Adjusted | | | | | |  |
| --- | --- | --- | --- | --- | --- | --- | --- |
|  | Inpatient Cost | | Pharmacy Cost | | Total Cost | | |
| Antibiotic Therapy | Model  OR (95%CI) | p-value | Model  OR (95%CI) | p-value | Model  OR (95%CI) | p-value | |
| Carbapenems | Model Not Significant |  | **0.66 (0.08-1.23)** | **0.026** | Model Not Significant |  | |
| Aminoglycosides | Model Not Significant |  | Model Not Significant |  | Model Not Significant |  | |
| Polymyxins | Model Not Significant |  | **1.32 (0.13-2.51)** | **0.029** | Model Not Significant |  | |
| Fluoroquinolones | Model Not Significant |  | Model Not Significant |  | Model Not Significant |  | |
| Ex. Spec. Cephalosporins | Model Not Significant |  | Model Not Significant |  | Model Not Significant |  | |
| Penicillins - β-lactamase inhibitor combinations | Model Not Significant |  | **-0.63 (-1.23- -0.03)** | **0.041** | **1.36 (00.61-2.12)** | **0.000** | |
| Tetracyclines | Model Not Significant |  | Model Not Significant |  | Model Not Significant |  | |
| Sulfonamides | Model Not Significant |  | Model Not Significant |  | **-4.01 (-5.96- -2.06)** | **0.000** | |
| Glycylcyclines | Model Not Significant |  | Model Not Significant |  | Model Not Significant |  | |
| Combination therapy | Model Not Significant |  | **0.77 (0.18-1.37)** | **0.011** | Model Not Significant |  | |
| Intra+extra therapy | Model Not Significant |  | Model Not Significant |  | Model Not Significant |  | |

Significant associations are shown in bold
